# Comparison of Anticancer Activity of *Dorycnium pentaphyllum* Extract on MCF-7 and MCF-12A Cell Line: Correlation with Invasion and Adhesion

**DOI:** 10.3390/biom11050671

**Published:** 2021-04-30

**Authors:** Gözde Koygun, Emine Arslan, Gökhan Zengin, Giustino Orlando, Claudio Ferrante

**Affiliations:** 1 Department of Nanotechnology and Advanced Materials, Selcuk University, Konya 42130, Turkey; gozdekayadibi@hotmail.com; 2 Department of Biology, Science Faculty, Selcuk University, Konya 42130, Turkey; gokhanzengin@selcuk.edu.tr; 3 Department of Pharmacy, Università degli Studi “Gabriele d’Annunzio”, Via dei Vestini 31, 66100 Chieti, Italy; giustino.orlando@unich.it (G.O.); claudio.ferrante@unich.it (C.F.)

**Keywords:** *Dorycnium pentaphyllum*, cancer, MCF-7, MCF-12A, invasion, bioinformatics

## Abstract

*Dorycnium pentaphyllum* subsp. *haussknechtii* is an important medicinal plant in several countries, including Turkey. This study aimed to evaluate the cytotoxicity of a crude extract of *D. pentaphyllum* subsp. *haussknechtii* against different breast cell lines to determine invasion, adhesion, and lipid peroxidation. The cytotoxic effects on MCF-7 breast cancer and MCF-12A as the immortalized cell line were examined by the XTT assay. Invasion and adhesion studies were performed according to the manufacturer’s kit procedure to IC_50_ values for 48 h. Lipid peroxidation was measured in the MCF-7 cell. A bioinformatics analysis was conducted to unravel the mechanism of action underlying antiproliferative effects, as well. According to XTT results, the tested extract showed a time- and a concentration-dependent cytotoxic effect. The most effective concentration was 100.5 µg/mL (48 h), which was selected for biological activities, such as apoptotic activity, invasion, adhesion, and lipid peroxidation assays. The extract caused tumoral cell death, and it did not have a cytotoxic effect on healthy human breast cells. Duplication times and measurement of CI analyses of cells were performed using the real-time cell analysis system xCELLigence. Finally, the bioinformatics analysis indicated the prominent role of quercetin as an extract component exerting a key role in the observed antiproliferative effects. This was supported by the micromolar/submicromolar affinity of quercetin towards proto-oncogene serine/threonine–protein kinase (PIM-1) and hematopoietic cell kinase (HCK), both involved in breast cancer. Altogether, our findings proposed that the extraction of the plant can be an effective strategy to isolate biomolecules with promising cytotoxic effects against breast cancer cells.

## 1. Introduction

It has been estimated that 3.5 million cancer-related deaths are annualized worldwide [1]. Although chemotherapy aims to kill cancer cells from a patient’s body, on the other hand, it also affects normal and healthy cells causing severe side effects, which in turn, results in multiple organ dysfunction [2,3]. Breast cancer is one of the most prevalent types of cancer in women all over the world. Along with lung and cervix cancers, this type of cancer is one of the most common types worldwide. According to statistics, one out of every eight women has breast cancer at certain times of her life. Treatment options may include one or more, depending on the stage of the disease, the characteristics of the patient, and the general health. In addition to methods, such as surgery, radiation therapy, hormone therapy, chemotherapy, targeted therapy, or herbal treatments are very popular and are continuing to develop rapidly [4]. The generation of invasiveness in transformed cells represents a required step of carcinoma progression [5]. Tumorigenic cell invasion is accompanied by various changes in cellular phenotype, which includes alterations in morphology, proteolytic activity and motility of the invasive cells [6]. Furthermore, a dramatic increase in the mobility of transformed epithelial cells can be observed during the adhesion test of carcinoma cells, contrasting with the low mobility of cells in normal epithelia.

In these, cell migration is restricted by the different kinds of junctional organelles, which tightly bind the cells together and thus immobilize individual cells. Thus, disruption of intercellular adhesion may be a prerequisite for releasing carcinoma cells from the epithelia and a key event during progression towards a fully malignant invasive cell [7].

Oxidative stress can occur in almost any tissue, and oxidative stress is known to play a master role in carcinogenesis [8]. In the normal biological process, there is a delicate balance between the formation of reactive oxygen species (ROS) and antioxidant substances. Low ROS level has a protective role in the cell under physiological conditions. However, the high accumulation of ROS within the cell can result in oxidative stress, leading to damage, such as genomic instability, and neoplastic transformations occur in DNA, thus leading to cancer [9,10]. During oxidative stress, superoxide anions, hypochlorite radicals, singlet oxygen, hydrogen peroxide, hydroxyl radicals, nitric oxide radicals, and lipid peroxides all increased in the cell. These increased substances react with membrane lipids, nucleic acids, proteins, enzymes and other small molecules to produce cellular damage [11]. Natural products have long represented a valuable source of compounds with promising applications in cancer chemotherapy due to their medicinal effectiveness, low toxicity, and many natural anticancer agents derived from different medicinal plants [12,13,14,15], that are usually rich in phenolic compounds, which could mediate, albeit partially, traditional and innovative pharmacological applications [16]. Chemoprevention by renewable phytochemicals could also be considered low-priced, quickly feasible, admissible, and sensible to cancer control and management. In the last years, research also deals with studying biochemical and therapeutic routes, appertaining to prevalent approaches due to natural anticancer agents in clinical practice, new candidate oncological therapy drugs from herbals, microorganisms, and promising chemopreventive agents from them [17].

*Dorycnium pentaphyllum*, a perennial herb of 10–80 cm height belonging to the *Dorycnium* genus of the *Fabaceae* family, is commonly found in central and southeast Europe [18]. Ethnobotanical data indicated that *D. pentaphyllum* was used as an anti-hemorrhoidal [19] and antidiarrheal [20]. *D. pentaphyllum* was previously reported to exhibit antibacterial activity [21]. *D. pentaphyllum* was also found to inhibit PGE_2,_ an inflammatory mediator, in human monocytes [19]. Studies have reported the presence of kaempferol, quercetin, myricetin [22], dorycnioside, kaempferol 3-O-(6″-acetyl)-β-glucopyranoside, (+)-dihydromyricetin, (−)-catechin, β-sitosterol, lotaustralin, gallic acid methyl ester, tachioside, d-pinitol, and phenylbutanone derivatives [18] in *D. pentaphyllum.* Many studies have investigated the antimicrobial, antibiofilm, and antioxidant potentials of *D. pentaphyllum* [23,24]. In the literature, two recent papers observed on cytotoxic properties of *D. pentaphyllum* extracts. Demir et al. [25] were reported the cytotoxic effect of this plant on human breast, liver, and lung cancer cells, and they reported IC_50_ values as 100.4 to 298.5 μg/mL in the tested cancer cell lines. In second study conducted by Demir et al. [26], the cytotoxic effect was investigated on the human cervix (HeLa), and colon (WiDr) cancer cells and IC_50_ values were found to be 46.5 and 84.5 μg/mL, respectively. However, there is still lack in scientific literature about the cytotoxic, antiproliferative effects and mechanistic approaches of this plant for inhibiting tumor growth of various cancer cell lines, including breast.

In this regard, in the present study, we aimed to investigate the anticancer effect and mechanism of action of *D. pentaphyllum* extract against human breast carcinoma cell line (MCF-7) and compare the effects with the immortalized breast cell line (MCF-12A). Most cancer cell types are thought to have improved adhesion ability, making it easier for cells to migrate to a new site to create new tumors in the body. In addition, we preferred to use adhesion and invasion assays, which can also be used to assess the effect of the extract treatments on the MCF-7 cell line to mimic the metastatic ability of cancer cells. The results of the cell adhesion assay permitted us to examine the interactions between the cells and extracellular materials herein. Finally, bioinformatics analysis was conducted to unravel the mechanism of action underlying antiproliferative effects.

## 2. Materials and Methods

### 2.1. Preparation of Plant Extract

The material (aerial parts of *D. pentapyllum* subsp. *haussknechtii*) for the extraction was collected in the area of Kahramanmaraş (Andırın) (Turkey) in summer 2015. Taxonomic identification was performed by the botanist Dr. Murad Aydin Sanda (Mus Alpaslan University, Department of Molecular Biology, Mus, Turkey). The grinding of naturally dried aerial parts of the plant was carried out by a mill in a laboratory. The extract was obtained by Soxhlet apparatus, and the procedure was given in our earlier paper [24]. The batch-to-batch consistency of the extract was being realized by mixing three different batches of extract.

### 2.2. Cell Culture

Immortalized human breast cell line, MCF-12A (ATCC^®^ CRL-10782™), was grown in DMEM-F12 medium (ThermoFischer, Waltham, USA) supplemented with 10% FBS (fetal bovine serum), 0.1% pen-strep, 1% NEAA (nonessential amino acids), 20 ng/mL of EGF(epidermal growth factor), 500 ng/mL of hydrocortisone, and 0.01 mg/mL human insulin.

The MCF-7 breast cancer cell line (ATCC^®^ HTB-22™) was obtained from American Type Culture Collection (Rockville, MD, USA). They were cultured with RPMI-1640 (BioChrom, Berlin, Germany) medium containing 10% FBS (Fetal Bovine Serum Biological Industries) (Sigma Aldrich, Milan, Italy) and 0.1% gentamicin sulfate solution (Sigma Aldrich, Milan, Italy). Both cell lines were kept under sterile conditions at 37 °C with 5% CO_2_ and 95% air, also subcultured weekly using 0.02% EDTA and 0.05% trypsin. Cells proliferating in cultures gradually lose their proliferation rate after consuming a high rate of nutrient agents in the cell culture medium or using the entire surface in which they can reproduce, and cell growths and deaths occur over time. In this period, the division of culture is the most accurate process to be done, and this process is called passaging [27]. The media were changed every 2–3 days.

### 2.3. Detection of Cell Viability by XTT Assay

The cell proliferation was determined by using a standard 2,3-bis [2-methoxy-4-nitro-5-sulfophenyl]-2H-tetrazolium5-carboxanilide inner salt (XTT) assay kit, which measures the metabolic activity of viable cells. In brief, the tested extract was horizontally diluted, respectively, from a high dose (5000 g/mL) to a low dose (9.75 low µg/mL). The extract was dissolved in DMSO (1%), and the control group contained just DMSO (without extract). MCF-7 and MCF-12A cells (1 × 10^5^ cells/well) were harvested from the exponential phase maintenance cultures, counted via Trypan blue exclusion assay, and seeded in 96-well plates in 50 µL volume per well by using a multichannel pipette. These procedures were repeated for 24, 48 and 72 h to see the changing of the extract how the effect on cell cultures. The assay is based on the cleavage of the tetrazolium salt XTT to form an orange soluble formazan dye by mainly mitochondrial dehydrogenase activity in living cells. Formazan formation was quantified spectrophotometrically at 490 nm using an ELISA reader (Biotek, Swindon, UK). Four replicates of each concentration were used in each assay and repeated three times. The results were compared and evaluated in terms of the response of breast cancer cells (MCF-7) and immortalized human cells (MCF-12A) for treatments.

### 2.4. RTCA System Measurements

By trypsinization, cells were harvested from exponential phase cultures, counted and then plated in 48-well plates. Sub-concentrations were prepared in serial dilutions from the stock solution. 50 µL of the medium was added to the e-plate wells. The cells used (MCF-7/MCF-12A) were inoculated in 50 µL of medium to contain 10^4^ cells in 50 µL, and the e-plate was incubated at room temperature for 30 min. Then the e-plate was placed in the plate area of the real-time cell analysis system (RTCA) device in the incubator. When the cell index (CI) measured by the device reaches 1, add different concentrations of the extract (assay concentration 100 µg/mL; 50 µg/mL, 25 µg/mL, and 12.5 µg/mL to the wells). 50 µL of medium containing 1 mL) was added. Just medium (with serum) was added to the control wells. After adding the tested extract, the experiment followed until the logarithmic phase on the CI-time graph was completed, and the CI values were measured at this time. To clarifying the cells’ response to the extract, the cells were observed every 60 min after the addition of the extract. Data analysis was performed with the RTCA software on the computer of the xCELLigence system. Each concentration was examined 3 times within the same experiment. General cell culture conditions and culture medium used for this method were similar to those applied for the xCELLigence counterpart experiments and applied cell densities (10,000 cells/cm^2^). Cell doubling time was calculated from the exponential phase of the growth curve.

### 2.5. Matrigel Invasion Assay

An invasion assay was treated by using 24well (8 μm pore size) Transwell plates (EZcell^TM^ cell invasion assay kit; Corning, Inc. Corning, NY, USA). MCF-7 cells were plated in the upper chambers in RPMI 1640 (serum-free) media at 15 × 10^4^ cells/well, which were pre-coated with collagen I., while the extract was maintained in the bottom chamber in RPMI 1640 (serum-free). After 48 h of incubation, MCF-7 cells were detected by crystal violet staining at room temperature for 30 min, and all the results were observed using inverted light microscopy (magnification, ×10 and ×40). Carefully aspirate the medium from the inside of the insert and gently swab the interior of the inserts to remove non-invasive cells. After cleaning the chambers, we added 400 µL of cell stain solution and incubate for 10 min at room temperature. For each well invasive cells were counted with a light microscope under a high magnification objective.

### 2.6. Adhesion Assay

MCF-7 breast cancer adhesion assay was performed by using a Vybrant^™^ cell adhesion assay kit (V-13181; Life Technologies, Grand Island, NY, USA) as described by the manufacturer. We confirmed this correlation by examining the MCF-7 breast cancer, which was treated with different concentrations of the tested extract and control groups. Briefly, 5 × 10^5^ MCF-7 breast cancer cells were seeded into a 96-well plate for two days. Cells were treated with or without extract at a 50% growth inhibitory concentration (IC50); 100.5 μg/mL. Also, 25 and 50 μg/mL concentrations of the extract were chosen where the cell viability was higher than the percentage of seventy. After 48 h incubation, MCF-7 cells were labeled with calcein-AM. They were co-cultured with the breast cancer cells for 2 h at 37 °C. After the indicated period, non-adherent cells were removed, and then calcein-AM measured the fluorescence using a fluorescein filter set (calcein has an absorbance maximum of 494 nm and an emission maximum of 517 nm) to calculate the number of adherent cells.

### 2.7. Lipid Peroxidation

Two different procedures were used for lipid peroxidation analysis. First, MDA (malondialdehyde) determination: The determination of MDA, an in vitro marker of lipid peroxidation, was determined according to the method proposed by [28]. According to this method, MCF-7 cells were treated with extract for 48 and 72 h, and 1 M HCl was added to cell lysates, followed by the addition of thiobarbituric acid (TBA) reagent and the resulting mixture was incubated at 95 °C. At the end of incubation, the absorbance of MDA was measured at 535 nm and 600 nm. The results obtained were corrected by subtracting nonspecific turbidity at 600 nm. Data are expressed as nmol/mg protein [28].

The second method, reduced glutathione (GSH) determination: GSH analysis, was performed using the method proposed by [29]. In this method, all proteins containing sulfhydryl in the cell lysates were precipitated with precipitation solution. The resulting sulfhydryl groups were treated with Ellman’s reagent [DTNB (5,5′-2-dithiobis nitrobenzoic acid)], and the absorbance of the resulting yellow color at 412 nm was measured. The values obtained will be expressed in mg/100 mL [29].

### 2.8. Bioinformatics

Putative targets were identified according to the bioinformatic method recently described [30,31]. Briefly, human proteins targeted by the extract phytocompounds were predicted using bioinformatics platform STITCH. Docking calculations were conducted through the AutoDock Vina of PyRx 0.8 software. Crystal structures of target proteins were derived from the Protein Data Bank (PDB) with PDB IDs as follows: 3BGZ (proto-oncogene serine/threonine–protein kinase (PIM-1)), 1QFC (hematopoietic cell kinase (HCK)). Discovery studio 2020 visualizer was employed to investigate the protein–ligand non-bonding interactions.

### 2.9. Statistical Analysis

Statistical significance between the control and treated MCF-7 cells and MCF12 A human immortalized cells were evaluated by using Student’s *t*-test to determine differences between mean values for normal distribution. All analyzed results were calculated with GraphPad Prism 6 Software (San Diego, CA, USA); a probability level <0.05 was considered significant throughout the analysis. Experiments were repeated at least four times. Continuous variables are given as mean ±1 standard deviation (SD).

## 3. Results

### 3.1. Cytotoxicity Assay on MCF-7 and MCF-12A Cells

**Figure 1:**
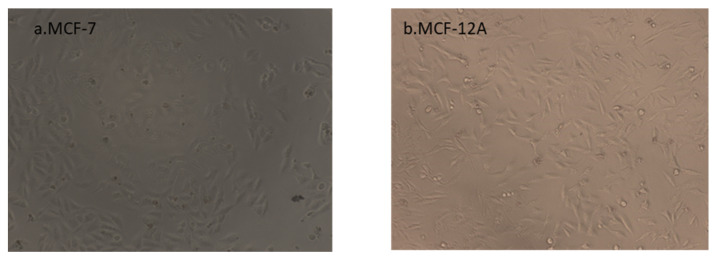
(**a**) Microscopic view of MCF-7 breast cancer cell cultures. (**b**) MCF-12A immortalized human breast cell cultures (Leica, 10× objective).

**Figure 2:**
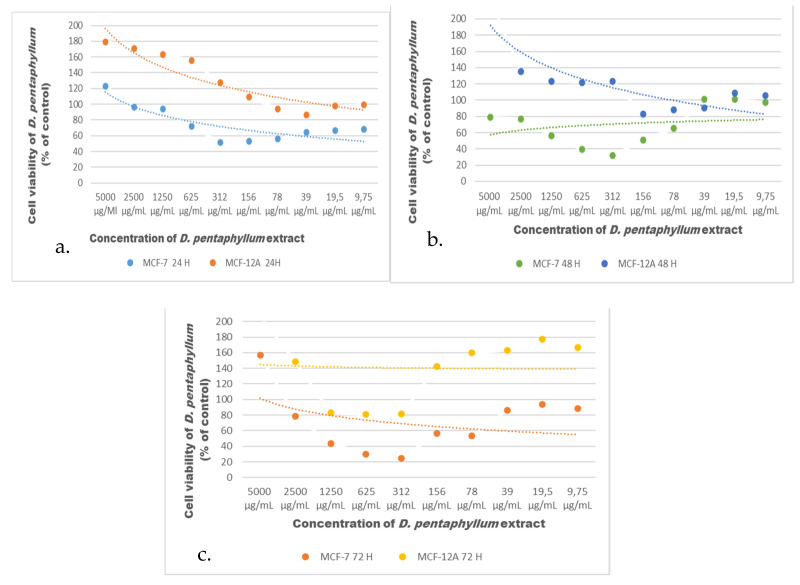
Effect of the extract on the viability of MCF-7 and MCF-12A cells assessed with the XTT assay after (**a**) 24, (**b**) 48 and (**c**) 72 h of treatment graph.

Figure 1a,b the MCF-7 breast cancer and MCF-12A breast cell lines, which were exposed to the extracts to evaluate antiproliferative effects. Specifically, the XTT test was used to determine the cytotoxicity effects of the extract in the range 9.765–5000 μg/mL. As shown in Figure 2, after 24 h of exposition to extracts, the extract anti-proliferative effect was evident in MCF-7 cells with an IC_50_ value of 78.69 μg/mL. In MCF-12A cells, the extract did not determine a viability fall below 80%, thus resulting biocompatible (*p* < 0.0001). After 48 and 72 h of treatment, the XTT results showed that the extract IC_50_ value was 100.5 μg/mL and 20.08 μg/mL, respectively. On the other hand, no toxic effect was observed in MCF-12A cells after 48 and 72 h exposition to the extract.

The results of the XTT assay showed that the extract induced time- and concentration-dependent cytotoxic effects on the MCF-7 cell line, whereas MCF-12A cells were unaffected by any concentration of the extract.

### 3.2. RTCA System Results

**Figure 3:**
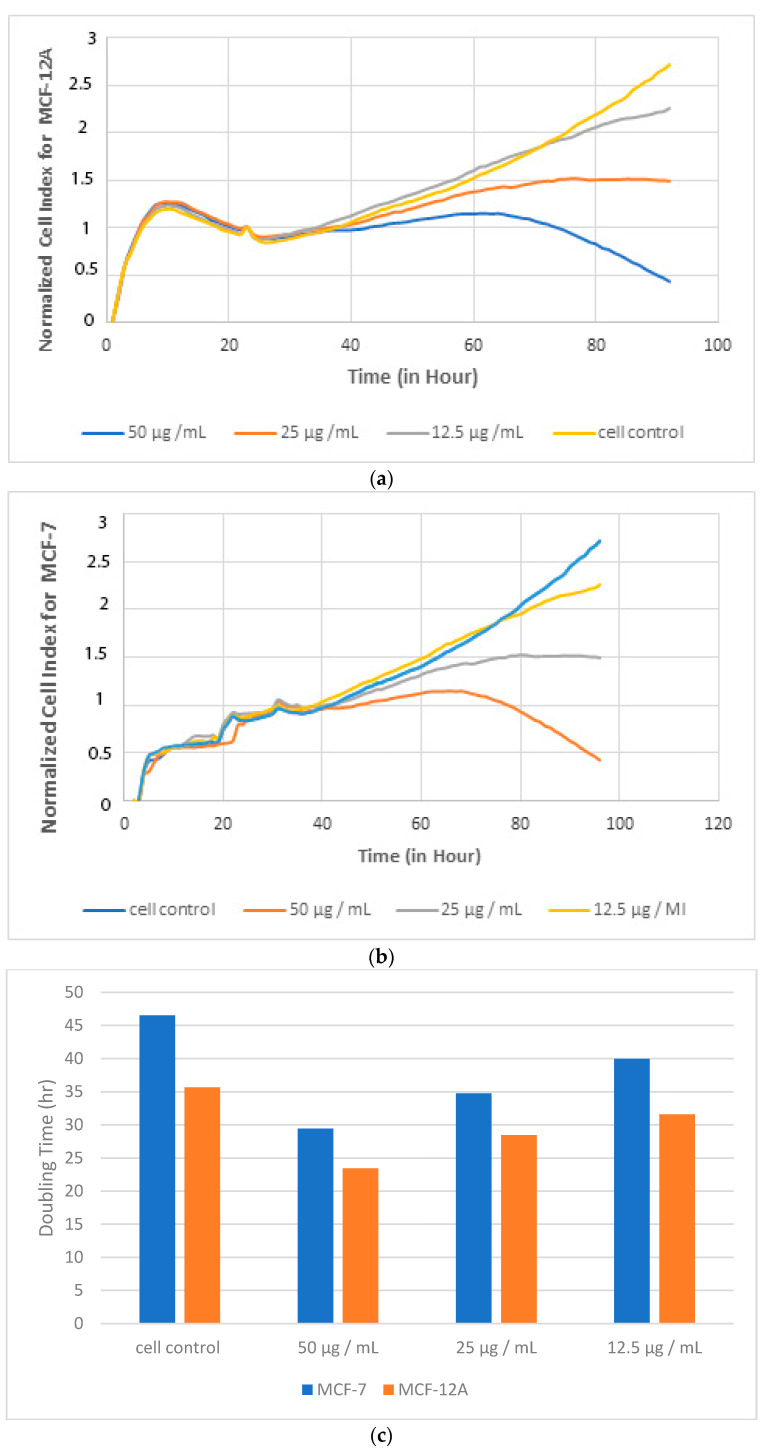
Normalized cell index (**a**,**b**) and duplication graph (**c**) of the effect of *Dorycnium* on MCF-7 breast cancer cells and MCF12 A human immortalized cells.

After adhering to the cells-plate surface, different concentrations of the extract prepared in the medium were applied to the cells and followed for 3 more days. In Figure 3a, the graph showing the duplication time of the extract is given on the MCF12 A with immortalized cell lines and on the MCF-7 breast cancer cell line in Figure 3b.

In cell culture studies with MCF-7 and MCF-12A cells models, *Dorycnium* extract concentrations less than 50 μg/mL (50, 25, and 12.5 μg/mL) were found to be safe in terms of cytotoxicity and duplication time. All data recorded using the xCELLigence RTCA system were processed using MS Excel to obtain data series with the same resolution. This was performed by selecting only the xCELLigence-generated values corresponding to the time points used in the reference methods, thus leading to a reconstruction of the studied process dynamics at a lower time resolution.

Based on the obtained graphs, the tested extract exhibited a cytotoxic effect with a concentration of 50 µg/mL and above. The concentrations of 12.5 and 25 µg/mL did not exhibit cytotoxic effects, and these concentrations were the same as control. In addition, duplication times were calculated for each cell line, and the results are given in Table 1. According to the duplication times, <25 µg/mL concentrations were found as safe on the MCF-12A cell line.

### 3.3. Role of the Extract on MCF-7 Breast Cancer Cells for Cell Differentiation and Invasion

**Figure 4:**
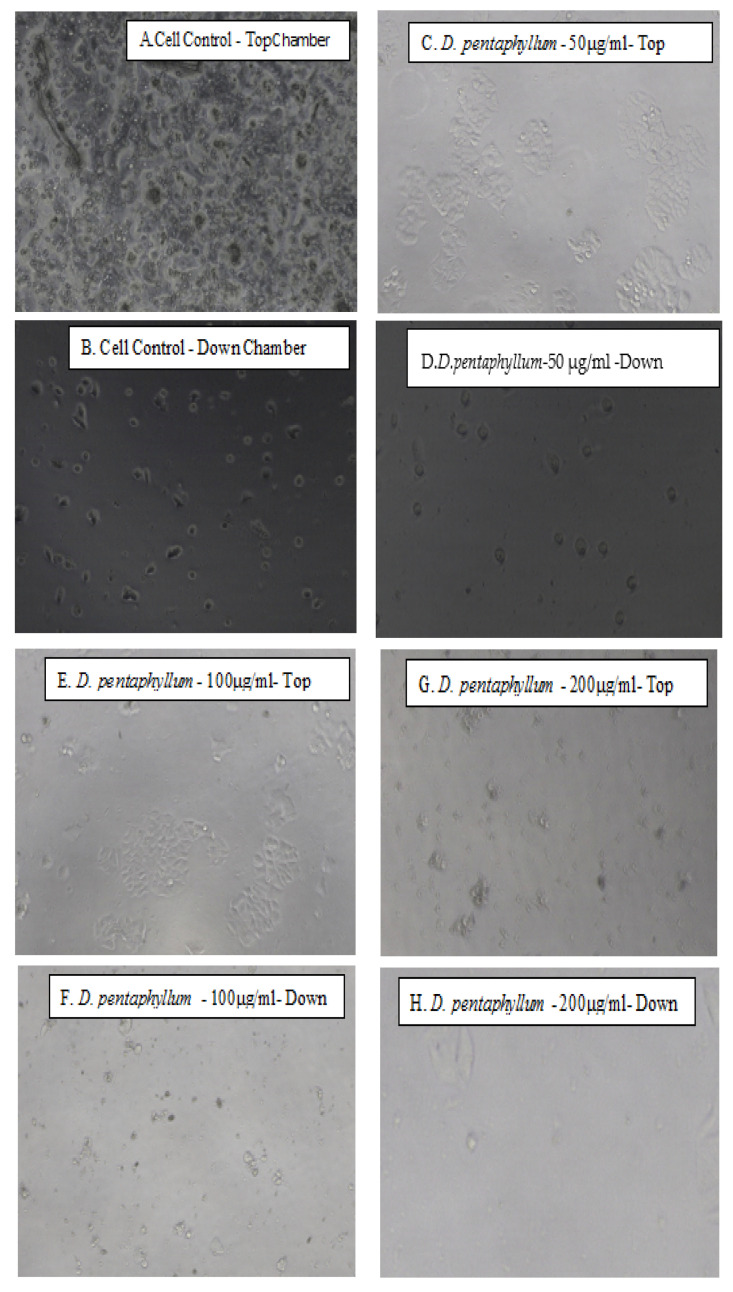

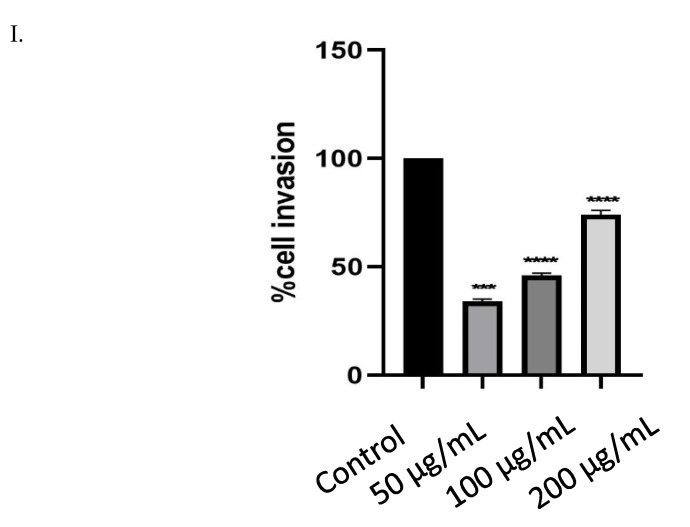
Cells were then incubated for 48 h. Photographs were taken at 0 h (**A**,**C**,**E**,**G**), and 48 h (**B**,**D**,**F**,**H**) using inverted microscope with 10× magnification and the fluorescent microplate reader absorbance invasion results of MCF-7 cells treated with different concentration of *Dorycnium* extract (**I**) **** *p* < 0.0001, *** *p <* 0.001 vs. control group (CC).

MCF-7 control group cells are highly invasive compared with MCF-7 cells with different concentrations of the extract. An EZ TM cell invasion (Matrigel) assay was used to determine the invasion potentials of the breast cancer cell line with the extract. Cells were seeded into either a porous 8 μm membrane insert (control) or Matrigel-layered membrane insert and incubated over its 50% growth inhibitory concentration (IC_50_) and two more different doses of extract concentrations for 48 h. Cells were fixed on the membrane, stained, photographed, and counted. Representative images of cells that invaded through the Matrigel layer (Figure 4) three concentrations of the extract were counted and graphed for cell invasion assay.

We characterized the invasive phenotype of MCF-7 cells by comparing them to MCF-7 cells with three different extract concentrations to the control group. The highest percentage of cell death for the extract was observed after 48 h incubation, and its 50% growth inhibitory concentration (IC_50_) was 100.5 μg/mL. The extracts were pretty effective on the MCF-7 breast cancer cell line. 100 µg/mL extract-treated MCF-7 breast cancer cell line was observed to be highly invasive compared to the MCF-7 control group. The effect of the extract (100 µg/mL) (IC_50_value) concentration results was found to be 5-times less invasive for collagen gels than the control group.

### 3.4. Effect of Cell Adhesion Secretion on MCF-7 CELLS

**Figure 5:**
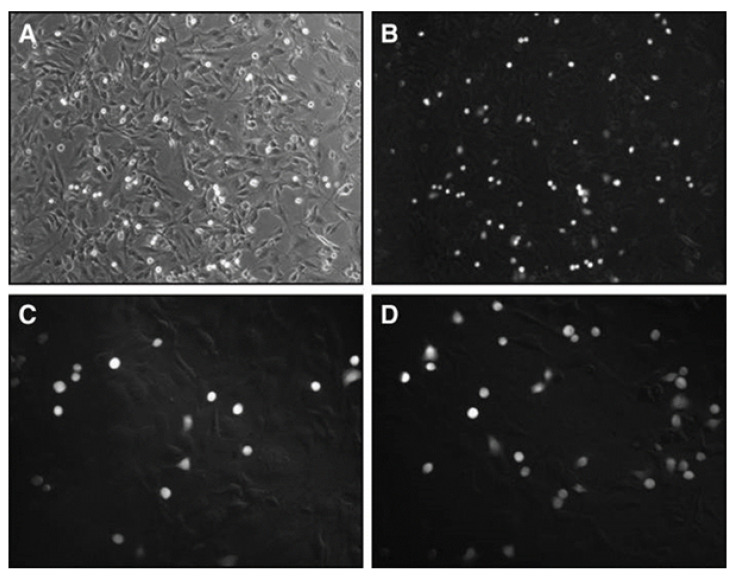
Representative photomicrographs of tumor cell adhesion.(**A**) MCF-7 cell control group. The Vybrant™ cell adhesion assay was used to assess the effect of the quantity of how dilutions of the tested extracts (**B**) 200 µL, (**C**) 100 µL and (**D**) 50 µL) effected on MCF-7 cells seeded on the number of adherent cells at 48 h incubation.

The ability of malignancy to invade and metastasize largely depends on the degree of epithelial differentiation within the tumors, i.e., defectively segregate being more invasive than well-segregated malignant tumor [32]. The conditions for MCF-7 cell adhesion assay were examined in 96-well cell culture treated with different concentrations of the extract in microplates for an effort to determine rapid, cost-effective methods to identify potential therapeutic compounds. After harvesting, dilutions of the extract (200 µL, 100 µL and 50 µL) were seeded in MCF-7 cell cultures per 96 wells, respectively. The Vybrant™ Cell Adhesion Assay was used to assess the effect of the quantity of how the extract effected on MCF-7 cells seeded on the number of adherent cells at 48 h incubation. This suggests that decreasing the concentration of the extract MCF-7 cells seeded leads to an increased number of adherent cells (Figure 5).

**Figure 6:**
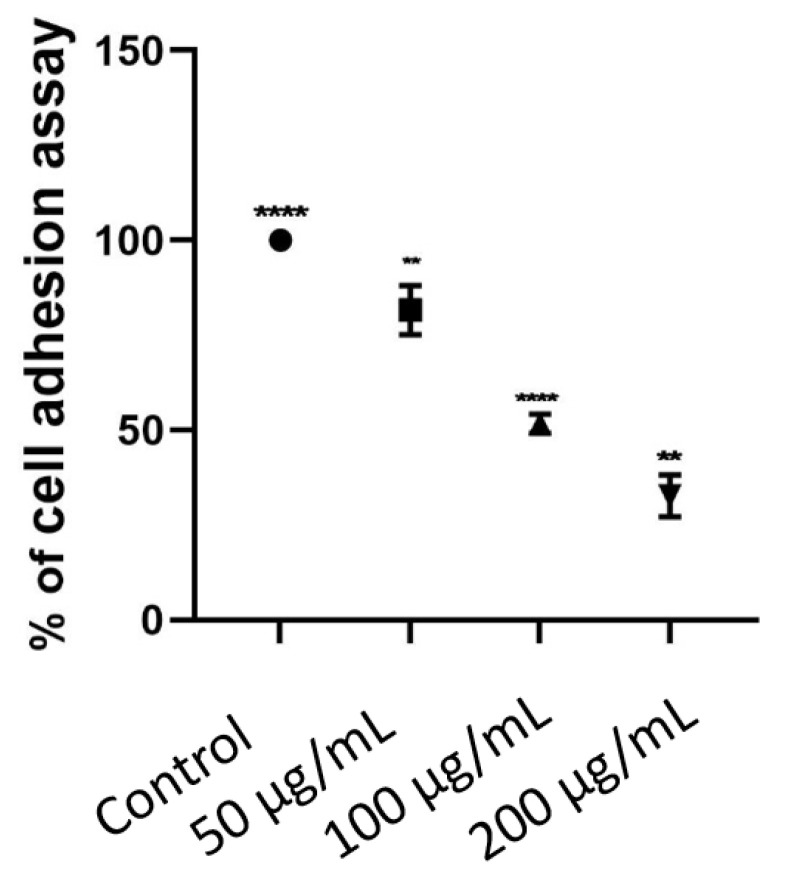
Cell adhesion results of the extract at concentrations of 50 μg/mL, 100 µg/mL, 200 μg/mL in MCF-7 cell line. Statistical analysis was performed using GraphPad Prism 9, using a two-way ANOVA with Tukey’s multiple comparison posttest. Only significant differences are displayed: ** *p* < 0.01, **** *p* < 0.0001.

Two fields of view were counted in each well (20× magnification), shown in Figure 6. Experiments were conducted a couple of times, both in triplicate. Our data showed that it expressed as the absolute number of cells adhered to the endothelial layer and as the percentage of cells adhered relatively to control.

In addition to evaluating adhesion for control and the extract applied to MCF-7 cells, assays were also performed at applying only tumor cells with the extract (200 µL, 100 µL and 50 µL) for 48 h (Figure 6).

As a result of adhesion analysis using the Vybrant cell adhesion analysis kit, application of the extract at concentrations of 50 μM, 100 μM 200 μM in MCF-7 cell line was decreased 82.4%, 50% and 27.2%, respectively compared to the control group. A significant result was determined at an IC_50_ concentration of 100 μM.

These findings indicate that using the extract can generate dedifferentiation and invasiveness of MCF-7 cells, and they suggest further that cytotoxic concentration of the extract acts as an invasion suppressor. In addition, quantification of tumor cell adhesion strength could operate as a predictor of the metastatic potential of a solid tumor. However, the obtained results must confirm by gene and protein expression analysis, and we strongly suggested that further analysis need a clear explanation of this case, and our findings could provide a light in the road.

### 3.5. Effect of the Extract on MDA and GSH Levels

**Figure 7:**
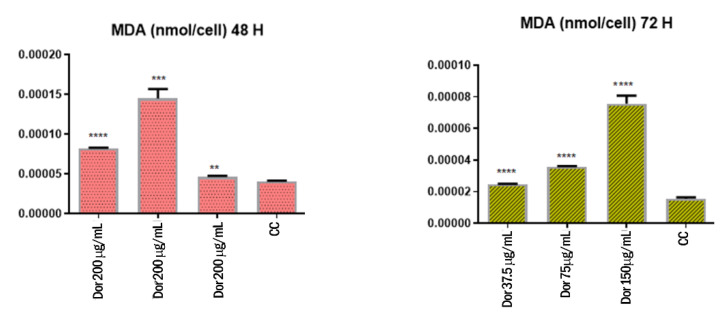
Effects of the extract (37.5–150 µM) on MDA levels in MCF-7 cells 48 and 72 h following treatment. **** *p* < 0.0001, *** *p <* 0.001, ** p < 0.05 vs. control group (CC).

**Figure 8:**
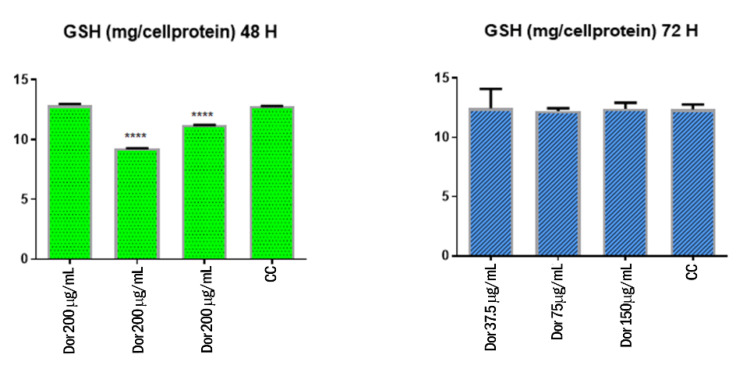
Effects of the extract (37.5–150 µM) on GSH levels in MCF-7 cells 48 and 72 h following treatment. **** *p* < 0.0001 vs. control group (CC).

Regarding the activity of extract on MDA and GSH levels in MCF-7 cells, it was observed a significant increase of MDA levels 48 and 72 h following treatment (Figure 7). However, the extract significantly reduced GSH 48 h following treatment, whereas the levels of GSH were unchanged compared to the control group 72 h after the stimulation (Figure 8).

### 3.6. Bioinformatics

**Figure 9:**
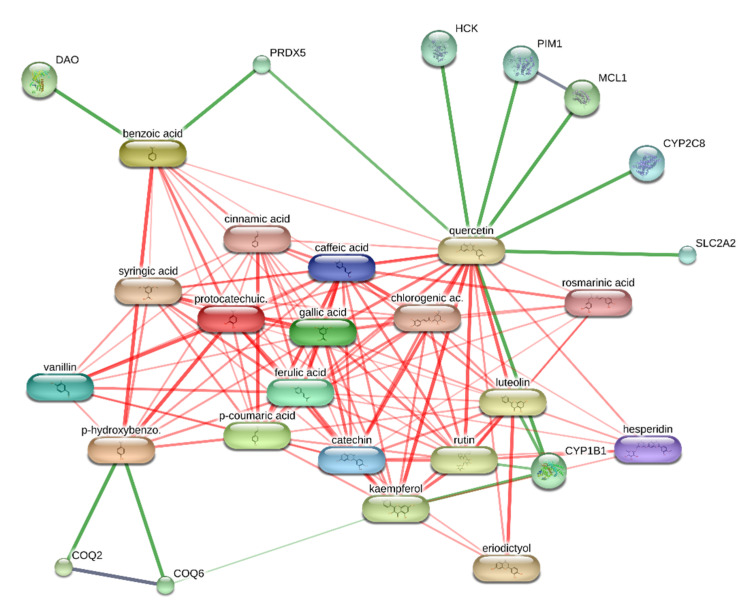
Components–targets analysis underlying the prominent position of quercetin, among all phytocompounds identified and quantified in the extracts. Noteworthy are the putative interactions towards proto-oncogene serine/threonine–protein kinase (PIM-1) and hematopoietic cell kinase (HCK), both involved in breast cancer.

**Figure 10:**
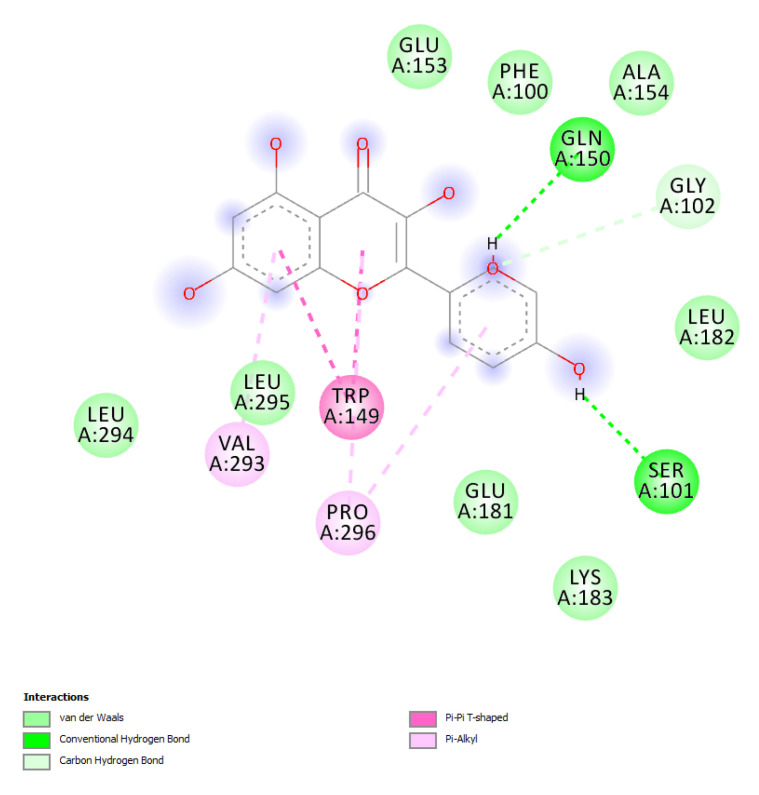
Putative interactions between quercetin and human and proto-oncogene serine/threonine–protein kinase (PIM-1; PDB: 3BGZ). Free energy of binding (ΔG) and affinity (Ki) are −7.4 kcal/mol and 3.8 µM, respectively.

**Figure 11:**
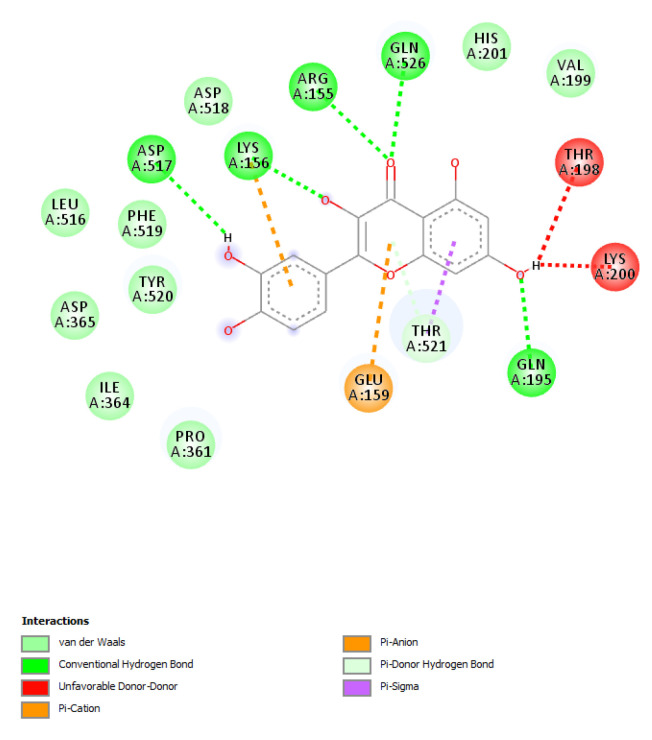
Putative interactions between quercetin and human and hematopoietic cell kinase (HCK; PDB: 1QFC). Free energy of binding (ΔG) and affinity (Ki) are −8.3 kcal/mol and 0.8 µM, respectively.

The results of the target prediction yielded by the STITCH platform are reported in Figure 8 and highlight the prominent position of quercetin in the network pharmacology analysis. It is interesting that quercetin was predicted to interact with PIM-1 and HCK proteins involved in breast cancer. The capability of quercetin to interact with these proteins was also demonstrated, albeit partially, by docking runs yielding affinity constants (Ki) in the range of 0.8–3.8 µM (Figure 9, Figure 10 and Figure 11).

## 4. Discussion

Herbal remedies play an important role in cancer prevention and treatment [33]. Anticancer study on MCF 7 cells showed that the death rate of cancer cell lines increases with a rise in the concentration of *Dorycnium* extract.

The *Dorycnium* genus is an important group of medicinal plants that have been employed in traditional medicine for a long time [19,26,34,35]. It is known that the plants are a significant source of flavonoids, and previous phytochemical investigations identified five flavonoids in the phytocomplex: myricitrin, quercitrin, kaempferol 3-O-β-glucopyranoside, kaempferol 3-O-(6″-acetyl)-β-glucopyranoside, (+)-dihydromyricetin [18].

Multiple studies conducted on the genus also showed antimicrobial, antibiofilm, and antioxidant activity, which were related, albeit partially, to the content of phenolic compounds [19,23,24]. However, the members of this genus have not yet been fully explored as cytotoxic. In this context, the present results contribute to a better understanding of their properties and potential application as antioxidant agents.

In our earlier paper [24], the chemical composition, antioxidant and enzyme inhibitory properties of the extract tested in the present study were determined. Catechin, quercetin and rutin were found to be the main phenolics in the chemical composition. From this point, the cytotoxic effects of the extract could be attributed to the presence of these compounds. Following our approaches, catechin exhibited significant anticancer and anti-lipid peroxidation abilities [36,37,38]. In addition, some authors reported that catechin induced apoptosis of several cell lines [39,40]. Similar findings were also observed for rutin [41,42] and quercetin [43]. Taken together, *D. pentaphyllum* subsp. *pentaphyllum* could be considered as a valuable source of anticancer compounds in the management of several cancer types.

Therefore, the cytotoxic effect of the tested extract was examined on MCF- 7 breast cancer cell lines and MCF12A immortalized cell lines using the XTT assay. In an earlier study, the effective antitumor effect has also been proven against HeLa and WiDr cells compared to normal fibroblast and colon cells from the *D. pentaphyllum* extracts. *D*. *pentaphyllum* extracts inhibited the cell viability of HeLa and WiDr cells in a concentration-dependent manner with lower IC_50_ values compared to normal colon and fibroblast cells. Furthermore, the IC_50_ values of the extract in HeLa cells were higher than cisplatin, but the selectivity index of the extract on HeLa cells (9.7-fold) was found higher than cisplatin (6.9-fold) [26].

Based on the results of the present study, it is confirmed that *D. pentaphyllum* extracts can be used as an alternative for the extraction of biomolecules for anticancer therapy.

Over 90% of human tumors are malignant tumors; in these, transformed epithelial cells grow in an uncontrolled fashion, break through the basement membrane, and invade the underlying mesenchyme [5]. According to this knowledge, we tried to figure out the invasiveness of MCF-7 breast cancer cells treated with the extract in vitro. Carcinomas can be subdivided by morphological and functional criteria: the first is that well-differentiated carcinomas retain epithelial tissue structures, which show well-developed intercellular junctions, and are generally weakly mobile and invasive. On the other hand, the second one is that poorly differentiated carcinomas are characterized by a more amorphous tissue structure, with fewer cell-to-cell junctions, thus resulting in more highly invasive [44]. In our study, 48 h application of the extract decreased (%) cell adhesion of MCF-7 breast cancer cells, thus further strengthening the importance of herbal extracts as sources of antiproliferative biomolecules against breast cancer [44,45,46].

Specifically, we used cell adhesion assay to see the extract affects the metastatic ability of MCF-7 breast cancer cells. So here, we wanted to show whether the cell adhesion was weakened on MCF-7 cells seeded on the number of adherent cells at 48 h incubation. As shown in Figure 4B, the number of adherent cells gradually decreased within a concentration-dependent manner, but the number of adherent cells treated with the tested extract for 48 h was significantly less than that of the control cells with significant statistical differences. This study provides the first evidence that the extract plays an important role in the development and progression of human cancer, MCF-7.

In the present study, the extract was effective, in the concentration range 37.5–150 µg/mL, to significantly increase the MDA levels in MCF-7 cells. Conversely, the GSH levels tend to decrease following extract treatment. Collectively, these findings showed pro-oxidant effects in MCF-7 cells, thus suggesting that the antiproliferative effects induced by the extract in this cell line could be the result of increased oxidative stress [45]. However, the pro-oxidant effect induced by the extract is contrasting with our previous observations of intrinsic/scavenging effects [24]. Nevertheless, we cannot exclude paradoxical pro-oxidant effects induced by the phenolic compounds present in the extract, particularly in vitro [46].

Based on the phytochemical composition of the extract, a bioinformatics investigation was conducted on the platform STITCH. The components-targets analysis considered all phytocompounds identified and quantified in the extracts and fully listed in Table 1. The results of the target prediction yielded by the STITCH platform highlight the prominent position of quercetin in the network pharmacology analysis. It is of noteworthy interest that quercetin was predicted to interact with PIM-1 and HCK proteins. In breast cancer, these target proteins could play pivotal roles in regulating cytokine signaling and cell proliferation, respectively [47,48]. The capability of quercetin to interact with PIM-1 and HCK was also demonstrated, albeit partially, by docking runs yielding affinity constants (Ki) in the micromolar range that agrees with the quercetin level in the extract. These results add to the observed inhibitory effects on MCF7 cell proliferation and lipid peroxidation induced by the extract, as well. Additionally, literature data showed the capability of quercetin to contrast breast cancer proliferation via multiple mechanisms, including the inhibition of lipid peroxidation and the modulation of apoptosis and inflammatory pathways [49]. To the best of our knowledge, this is the first in silico study suggesting the quercetin affinity towards PIM-1 and HCK proteins. In this regard, future studies are required to confirm these putative interactions via independent biochemical analyses.

## 5. Conclusions

Medicinal plants have a great place in cancer therapy and are a valuable resource for discovering small-molecule inhibitors that target signal transduction proteins, e., kinases, which regulate the proliferation and invasion of cancer cells. We are convinced that the inclusion of these assays in relevant quantitative methods will be a powerful tool for the preclinical screening of new therapeutic drugs designed to characterize cancer metastatic prognosis and disrupt metastatic progression. In summary, our data demonstrate the efficacy of *D. pentaphyllum* extract in inhibiting cell proliferation, invasion and adhesion, which could be related, albeit partially, to the quercetin content. This further adds to the importance of validating using traditional medicinal plants and herbs in breast cancer therapy.

## Figures and Tables

**Table 1:** MCF-7 and MCF-12A duplication time chart.

Well	Doubling Time (h) ± Standard Deviation (SD)
MCF-12A cell control	35.66 ± 3.5
MCF-12A—50 μg/mL	23.42 ± 0.37
MCF-12A—25 μg/mL	28.45 ± 2.72
MCF-12A—12.5 μg/mL	31.57 ± 3.80
MCF-7 cell control	46.53 ± 2.97
MCF-7—50 μg/mL	29.43 ± 0.74
MCF-7—25 μg/mL	34.74 ± 1.12
MCF-7—12.5 μg/mL	39.94 ± 1.54

## Data Availability

The data presented in this study are available on request from the corresponding author.
